# Identifying gaps in the practices of rural health extension workers in Ethiopia: a task analysis study

**DOI:** 10.1186/s12913-017-2804-0

**Published:** 2017-12-20

**Authors:** Firew Ayalew Desta, Girma Temam Shifa, Damtew WoldeMariam Dagoye, Catherine Carr, Jos Van Roosmalen, Jelle Stekelenburg, Assefa Bulcha Nedi, Adrienne Kols, Young Mi Kim

**Affiliations:** 1 Jhpiego, Addis Ababa, Ethiopia; 2 Jhpiego, 1615 Thames Street, Baltimore, MD 21231 USA; 3 Vrije Universiteit Amsterdam, Amsterdam, The Netherlands; 4 Medical Centre Leeuwarden, University Medical Centre Groningen/University of Groningen, Groningen, The Netherlands

**Keywords:** Task analysis, Community health worker, Health extension worker, Health extension program, Pre-service education, In-service training, Scope of practice, Ethiopia

## Abstract

**Background:**

Health extension workers (HEWs) are the frontline health workers for Ethiopia’s primary health care system. The Federal Ministry of Health is seeking to upgrade and increase the number of HEWs, particularly in remote areas, and address concerns about HEWs’ pre-service education and practices. The aim of this study was to identify gaps in HEWs’ practices and recommend changes in their training and scope of practice.

**Methods:**

A cross-sectional descriptive task analysis was conducted to assess the work of rural HEWs who had been in practice for six months to five years. One hundred participants were invited from 100 health posts in five regions of Ethiopia. HEWs self-reported on 62 tasks on: frequency, criticality (importance), where the task was learned, and ability to perform the task. Descriptive statistics, including frequencies and percentages, were computed for each variable. Task combinations were examined to identify tasks performed infrequently or for which HEWs are inadequately prepared.

**Results:**

A total of 82 rural HEWs participated in the study. Nearly all HEWs rated every task as highly critical to individual and public health outcomes. On average, most HEWs (51.5%–57.4%) reported learning hygiene and environmental sanitation tasks, disease prevention and control tasks, family health tasks, and health education and communication tasks outside of their pre-service education, primarily through in-service and on-the-job training. Over half of HEWs reported performing certain critical tasks infrequently, including management of supplies, stocks and maintenance at the facility and management of the cold chain system. Almost all HEWs (95.7–97.2%) perceived themselves as competent and proficient in performing tasks in all program areas.

**Conclusion:**

HEWs were insufficiently prepared during pre-service education for all tasks that fall within their scope of practice. Many learned tasks through in-service or on-the-job training, and some tasks were not learned at all. Some tasks that are part of expected HEW practice were performed infrequently, potentially reducing the effectiveness of the Health Extension Program to provide preventive and basic curative health care services to communities. Findings should alert policy makers to the need to review HEWs’ scope of practice, update pre-service education curricula and prioritize in-service training modules.

## Background

Like other sub-Saharan African countries, Ethiopia suffers from severe health workforce shortages. The health workforce density (including doctors, health officers, nurses and midwives) per 1000 population in 2014 was 0.7 [1], significantly lower than the minimum threshold of 2.3 health workers per 1000 (including doctors, nurses and midwives) recommended by the World Health Organization (WHO) [2]. In response to this critical shortage, Ethiopia like many other countries in Africa, Asia, and Latin America has invested heavily in community based primary health care to bring services to the rural and remote areas where most of the population lives [3]. In 2004 the Ethiopian Federal Ministry of Health (FMOH) launched a Health Extension Program, grouping 17 essential health services under four program areas: a) hygiene and environmental sanitation, b) family health services, c) disease prevention and control, and d) health education and communication [4].

Health extension workers (HEWs) now serve as the main frontline providers for the national health care system. They are salaried employees of the FMOH and provide preventive and basic curative health care services at rural health posts and manage needed supplies and equipment [3, 5]. They also receive other incentives, such as housing at some health posts. As of 2013, 38,000 HEWs were serving in 16,440 rural health posts [1]. Health posts are the operational unit of the Health Extension Program and are furnished with the equipment, materials and supplies required to deliver the different packages of essential services to the community. The deployment of HEWs has increased potential health service coverage from 64% of Ethiopia’s population in 2004 to 92.1% in 2011 [4]. However, the cadre has a high turnover rate and concerns have been expressed about HEWs’ preparation for practice and their performance [4, 6].

Since the start of the Health Extension Program, some relevant health indicators have improved while others have remained stagnant. For example, under-five mortality in Ethiopia declined from 166 per 1000 live births in 2000 to 88 per 1000 live births in 2011, but maternal mortality remained high at 676 deaths per 100,000 live births and only 34% of pregnant women received antenatal care (ANC) from skilled providers [7]. Although maternal, newborn and reproductive health services are within the scope of HEW practice, national estimates indicated that HEWs provided only 8.7% of ANC, 0.8% of clean and safe delivery care, and 0.4% of postnatal care services [7].

Rural HEWs, who are primarily women, must have at least a 10th grade education. They are selected from the communities where they live, which avoids language and cultural barriers and increases community acceptance. After one year of pre-service training, during which they receive a food and housing allowance. HEWs graduate with level 3 (certificate level) qualifications. The government initiated a continuing professional development scheme in 2012 to upgrade level 3 certificates to level 4 (diploma level) with one year of additional education at a technical college. HEWs continue to receive their regular salaries during level 4 training. HEWs must take both entrance and qualifying pre-post knowledge tests. Essentially, the post-test serves as a national qualifying exam for both levels unless HEWs pass it, they are not allowed to practice. A minimum of two HEWs, either level 3 or level 4, are stationed at each rural health post to serve a population catchment of 5000. HEWs are expected to work 25% of their time at the health post and 75% at community and household levels [3, 8]. After deployment, HEWs receive refresher trainings using five modules focused on the Health Extension Program. They also receive on-the-job training from their supervisors or senior experts. HEWs are supervised by the health center staff on technical issues and by district health office and Kebele (lowest administrative unit) staff for administrative purpose. HEWs are also supervised by FMOH staff and regional health bureau staff on quarterly and biannually.

A government sponsored evaluation of the Health Extension Program conducted in 2011 indicated that HEWs working at rural health posts provided ANC services for 80 pregnant women and conducted 25 deliveries each year, on average, but they lacked skills in performing tasks related to maternal and child health [5]. Similarly, a study of HEWs in three districts in the northern region of Tigray found they had poor knowledge of ANC counseling (54%) and difficulty identifying danger signs and complications of pregnancy (88%); nevertheless, most (92%) said they assisted labor and deliveries at health posts and in the home [9].

Given concerns about the quality of services provided by HEWs and a lack of detailed information about their work at the health post and community levels, there is a need for additional current information. This study employed the descriptive research methodology of task analysis to explore current practices of HEWs. Task analysis is a descriptive and analytic methodology that can be used to explore the practice of health workers for purposes of health system strengthening. Oshio and colleagues [10], p35 define task analysis as a “systematic assessment of the knowledge, skills and abilities (professional behaviors) that characterized clinical practice.” Task analysis has been used by health professions in North America to regularly update content of licensing examinations [11, 12]. In Africa, task analysis had been conducted with nurses and midwives to verify practice scope and standards for nurses in Lesotho [13], to assess the maternal-newborn nursing workforce in Mozambique [14], to identify gaps in education in Liberia [15], and to develop a licensing exam in Botswana [16].

The objective of this study was to gather information about the frequency, criticality, training, and performance of tasks by rural HEWs in order to identify gaps and recommend changes in their pre-service education, in-service training and scope of practice.

## Methods

### Study design

This study employed a cross-sectional design and task analysis methodology to assess the practice of recently graduated rural HEWs working at health posts from 6 months to 5 years.

### Sample size and sample selection

This study covered the five regions where 92% of Ethiopia’s population resides: Tigray, Amhara, Oromia, Southern Nations, Nationality and People (SNNP) and Somali. HEWs with less than 6 months of work experience were excluded from the study because of inadequate exposure to work demands. HEWs with more than 5 years of work experience were also excluded on the assumption that they might have poor recall of their educational preparation for specific tasks. Both level 3 and level 4 HEWs were included in the study because they have a similar scope of work. An estimated 4272 HEWs with 6 months to 5 years of work experience were serving in the target regions at the time of the study.

The purpose of the study was to do a quick assessment with as many as HEWs as possible within a limited budget, and those considerations drove the sampling approach. After consulting with experts in the field of task analysis, we decided that a sample of 100 HEWs drawn from heterogeneous locations across the five most populous regions in Ethiopia would provide reliable information at the aggregate level; the study was not designed to assess regional differences. We assumed that HEWs practices would be largely similar across and within regions because their scope of work and practice environment (the health unit) is the same. The sample was designed to include 20 HEWs from each of the five regions, with each HEW drawn from a different rural health post. To ensure the representativeness of the data, each of the five regional health bureaus sent recruitment letters to district health offices across the region, including in remote areas. The district health offices compiled a list of health posts with HEWs on staff who met the inclusion criteria and then randomly selected health posts to participate in the study. Two to four HEWs work at each health post. If more than one of them were eligible for the study based on the length of their work experience, the district health office randomly selected one of them to participate in a data collection workshop, assuming that all HEWs working in the same health post would be performing similar tasks with similar frequency. Limiting study participation to one HEW per health post also helped to avoid service interruptions at the sampled health posts. However, 18 of the 100 HEWs who came to the workshop had more than 5 years of work experience and had to be excluded from the study. Hence, the final sample included 82 HEWs from 82 health posts and was skewed towards certain regions.

### Data collection instrument

A task list was developed in collaboration with an expert panel from relevant health professions, ministries, and educational institutions in Ethiopia. The 30 experts on the panel included senior HEWs, nurses, midwives, sanitation specialists, and health officers. Reference documents used included occupational standards, job descriptions, curricula for HEWs, and national health sector development program plans from 2010 to 2015. The panel identified and approved 62 task items performed by both level 3 and level 4 HEWs in the four major areas covered by the Health Extension Program: hygiene and environmental sanitation (7 tasks), disease prevention and control (13 tasks), family health (22 tasks), and health education and communication (20 tasks).

The study questionnaire comprised basic demographic variables (gender, age, region, and years of work experience) and the list of 62 tasks with corresponding measurement variables. The measurement variables were frequency of task performance (how often did the HEW perform the task?), criticality or importance of the task (how critical is timely and effective performance of the task to patient/public health outcomes as perceived by the HEW?), education/training (how did the HEW learn to perform the task?) and self-perceived performance (how competent did the HEW believe s/he was in performing the task?). The response categories for frequency ranged from 1 to 5 (1 = never, 2 = rarely, 3 = monthly, 4 = weekly, and 5 = daily). Criticality included three response options: low, moderate and high criticality. Education/training had four possible responses: pre-service education, in-service training, on-the-job training, and never trained on the task. Performance ratings included three responses: not able to perform safely, competent, and proficient. These measurement variables were adopted from a Lesotho nursing task analysis study [13].

The questionnaire was translated from English to four local languages (Amharic, Oromifa, Tigrigna, and Somali languages) by a government-recognized translating firm.

### Data collection

Data collectors were health professionals who had experience in data collection and were acquainted with the Health Extension Program. In order to ensure fidelity to the study protocol, a data collector training was conducted to review the study questionnaire, ethical issues, data collection procedures and data quality assurance methods. Teams of data collectors and supervisors were deployed in the five selected study regions during a one-week period in December 2013. A total of 18 data collectors, supervisors and coordinators were involved in the study.

Participating HEWs attended a one day data collection workshop in their region’s capital city that was organized especially for the purpose of data collection. Each of the five workshops had a facilitator and a co-facilitator who served as data collectors. Verbal informed consent was obtained from each participant prior to data collection. HEWs completed the socio-demographic questions and rated each of the 62 tasks on all four measurement variables.

### Data entry and analysis

Completed questionnaires were sealed in envelopes and taken to the study’s research office in Addis Ababa. Data were double entered and analyzed using SPSS 23 [IBM SPSS statistics 23, http://www.ibm.com]. Data cleaning was done using frequency results in order to check inconsistencies and outliers.

Responses for measurement variables were combined within categories and coded to perform simple descriptive analyses. Frequency was categorized as high (weekly or daily) or low/infrequently (never, rarely, or monthly); criticality was categorized as high (moderate or high criticality) or low; education/training was categorized as pre-service education, not learned during pre-service education (in-service or on-the-job training), or never trained on the task; and self-perceived performance was categorized into competent (proficient or competent) or not able to perform safely.

Descriptive statistics, including frequencies and percentages were computed for each task measurement variable. Task combinations were examined to identify tasks that need special attention, which are defined as important tasks that HEWs perform infrequently or for which they are inadequately prepared. The 62 tasks were arranged in descending order of percentage values for each measurement variable in order to decide cut-off values for analysis of combinations. Initially we explored the data using the mean and median values to set cut-off points for further analysis. However, this approach omitted some important tasks. For example, tasks such as “provide essential newborn care services” and “manage cold chain system” had criticality values less than the mean. Therefore, cut-off values were determined subjectively by the research team for rankings of high criticality (>80%), infrequent performance (>50%), not able to perform safely (>3%), never trained on the task (>2%), and not learned during pre-service education (>50%) (Table 1).

**Table 1:** Combinations of task measurement variables used to identify tasks that need attention

Task combinations	Implications
High criticality + infrequently performed	Infrequency may be due to naturally rare occurrence or lack of training or infrastructure (e.g., not done in that setting). Depending on the reason for infrequency, tasks may be considered for development or revision of pre-service education (PSE) curricula or for in-service trainings for working HEWs.
High criticality + not able to perform safely	Provide in-service training, mentorship, and supervision for working HEWs and reconsider pre-service education.
High criticality + never trained	Tasks within the HEW scope of practice should be considered during development or revision of pre-service education curricula. Adding targeted in-service trainings provides the skills for working HEWs.
High criticality + not learned during pre-service education	Tasks need consideration during development or revision for HEW pre-service education curricula.

## Results

### Characteristics of the sample

Demographic characteristics of the HEWs in the sample are presented in Table 2. Most HEWs were female and less than age 30 years; their mean service duration was 2.3 years.

**Table 2:** Demographic characteristics of respondents (*N* = 82)

Variables	%	Mean	Range
Sex			
Male	13.4		
Female	86.6		
Age in years		24.9	19–42
< 20 year	3.7		
20–24 years	45.1		
25–29 years	43.9		
> 29 years	7.3		
Service duration in years		2.3	0.5–4.9
Region			
Tigray	23.2		
Amhara	20.7		
Oromia	12.2		
Somali	28.0		
SNNP	15.9		

### Description of tasks

Table 3 summarizes the ratings of the 62 tasks for each measurement variable, by the four Health Extension Program areas. On average, 74.2% of HEWs reported performing each hygiene and environmental sanitation task at high frequency. Health education and communication tasks were performed infrequently by about 40% of HEWs, on average. Almost 90% of HEWs considered the tasks in each health extension program area to be highly critical to individual and public health outcomes. Most HEWs (from 51.5% to 57.4%, on average) learned how to perform the tasks in each program area during in-service or on-the-job trainings rather than in pre-service education. Almost all HEWs considered themselves to be competent or proficient in performing the tasks in each program area, with the average ranging from 95.7% to 97.2%.

**Table 3:** Average percentage of HEWs who rated the tasks in a Health Extension Program area (N = 82)

Program area		Frequency		Criticality		Education/training			Self-perceived performance	
	Number of tasks	High	Low	High	Low	Learned in PSE	Not learned in PSE	Never trained at all	Competent or proficient	Not able to perform safely
Hygiene and environmental sanitation	7	74.2	25.8	88.8	11.2	43.6	54.2	2.3	97.2	2.8
Disease prevention and control	13	73.4	26.7	89.9	10.1	44.9	52.0	3.1	95.7	4.3
Family health	22	69.3	30.7	89.8	10.2	45.3	51.5	3.2	95.6	4.4
Health education and communication	20	60.4	39.6	89.5	10.6	39.8	57.4	2.8	95.7	4.3

### Task combination: Highly critical, but infrequently performed

There were five tasks that over 80% of HEWs categorized as highly critical to individual and public health outcomes, but that more than 50% of HEWs performed infrequently. Providers were most likely to report performing research activities (79%) and acting as clinical preceptors (66%) infrequently (Table 4).

**Table 4:** Tasks rated as highly critical that HEWs performed infrequently (N = 82)

	Percent of providers who rated the task as:	
Priority tasks	High criticality %	Infrequently performed %
Participate in research activities focusing on data management and reporting	80.4	79.0
Act as clinical preceptor, including facilitation of learning activities	87.8	66.0
Evaluate the effect of health information provided to community	91.5	57.0
Manage supplies stocks and maintenance at facility	86.6	55.0
Manage cold chain system	84.1	51.0

### Task combination: Highly critical, but perceived as not able to perform safely

There were 14 tasks that over 80% of HEWs categorized as highly critical to individual and public health outcomes, but that more than 3% of HEWs said they could not perform safely. More than one-fifth (22%) of HEWs said they could not safely provide Integrated Management of Newborn and Child Illness (IMNCI) or provide institutional clean and safe delivery services; 13.4% said they could not safely manage supplies stocks and maintenance at the facility (Table 5).

**Table 5:** Tasks rated as highly critical that HEWs were not able to perform safely (N = 82)

	Percent of providers who rated the task as:	
Priority tasks	High criticality %	Not able to perform safely %
Provide Integrated Management of Newborn and Child Illness (IMNCI)	90.2	22.0
Provide institutional clean and safe delivery services	90.2	22.0
Manage supplies stocks and maintenance at facility	86.6	13.4
Participate in research activities focusing on data management and reporting	80.4	9.8
Act as clinical preceptor, including facilitation of learning activities	87.8	8.5
Evaluate the effect of health information provided to community	91.5	7.3
Diagnose and treat minor uncomplicated diseases	84.1	6.1
Prevent and control non-communicable diseases	84.1	6.1
Conduct community diagnosis of communicable disease	92.7	4.9
Counsel, test & refer mothers for PMTCT	82.6	4.9
Establish and demonstrate community-appropriate sanitation technologies	89.1	3.7
Provide essential newborn care services	89.0	3.7
Provide Integrated Community Case Management (ICCM)	86.5	3.7
Manage cold chain system	84.1	3.7

### Task combination: Highly critical, but learned outside of pre-service education

There were 14 tasks that over 80% of HEWs rated as highly critical, but that less than half were trained on during pre-service education. In-service and on-the-job training were the most common learning paths for Integrated Community Case Management (72%) and occupational health safety management (69.5%). Substantial numbers of HEWs said they were never trained on IMNCI (22%) and institutional clean and safe delivery services (15.9%) (Table 6).

**Table 6:** Tasks rated as highly critical that HEWS did not learn during pre-service education (N = 82)

	Percent of providers who rated the task as:		
Priority tasks	High criticality %	Learned during in-service or on the job training %	Never trained at all %
Establish and demonstrate community-appropriate sanitation technologies	89.1	53.6	4.9
Establish and maintain occupational health safety management system	84.1	52.5	6.1
Conduct community diagnosis of communicable disease	92.7	50.0	3.7
Manage supplies stocks and maintenance at facility	86.6	53.6	11.0
Diagnose and treat minor uncomplicated diseases	84.1	53.6	3.7
Prevent and control non-communicable diseases	84.1	48.8	3.7
Counsel, test & refer mothers for PMTCT	82.6	52.5	2.4
Provide Integrated Management of Newborn and Child Illness (IMNCI)	90.2	52.4	22.0
Provide institutional clean and safe delivery services	90.2	48.8	15.9
Provide essential newborn care services	89.0	46.3	3.7
Provide Integrated Community Case Management (ICCM)	86.5	72.0	2.4
Manage cold chain system	84.1	50.0	8.5
Act as clinical preceptor, including facilitation of learning activities	87.8	64.6	4.9
Participate in research activities focusing on data management and reporting	80.4	54.9	11.0

## Discussion

This task analysis reveals a mismatch between rural HEWs’ pre-service training and their scope of practice. On average, less than half of HEWs reported learning the tasks in each performance area, including highly critical tasks and during pre-service education. For example, expanding case management of childhood illness services to the community level is a common strategy to reach more children and provide them with accessible lifesaving treatments [17]. Most of the HEWs in this study said they frequently performed Integrated Community Case Management (ICCM) and felt competent to do so, but the vast majority (72%) learned how to perform the task during in-service or on-the-job training. This is similar to a study in sub-Saharan Africa that reported most community health workers learned ICCM skills during in-service training [18].

In order to improve health outcomes, health workers’ scope of practice must drive pre-service education [19]. However, discrepancies between the two are not unique to Ethiopia. Task analyses in other countries have also found that the pre-service education curriculum does not necessarily cover health workers’ expected or actual scope of practice. For example, a study in Liberia found that neither pre-service nor in-service training covered core tasks for nurses and midwives related to antenatal care and family planning [20]. Similarly, pre-service education for medical licentiates in Zambia failed to cover anesthesia, even though the cadre felt obligated to offer the service in the absence of other qualified providers [21].

Notably, the HEWs in this study felt able to perform almost all of the tasks in the scope of practice despite the gaps in pre-service education; the findings highlight the importance of in-service and on-the-job training. This confirms previous studies in Ethiopia that found large numbers of HEWs learned their work through on-the-job training [5, 9]. However, it contradicts the accepted role of in-service training for frontline health workers like HEWs, which is to strengthen and build on skills first learned during pre-service education [22]. Although in-service and on-the-job training may be a reasonable approach to teach HEWs how to perform certain uncomplicated tasks, it is not appropriate when the safe performance of a task is critical to address health problems in the community, as is the case with ICCM. These critical tasks need to receive special attention during pre-service education to assure that HEWs learn them adequately; this may require revising the curricula to increase the focus on key tasks.

Another key finding from the task analysis is that HEWs in Ethiopia did not perform some highly critical tasks as regularly as they should. For example, around half of HEWs reported that they infrequently managed the cold chain system and supplies, stocks, and maintenance at the facility, even though immunizations are one of HEWs’ main responsibilities and these tasks ensure the availability and the viability of vaccines and other lifesaving drugs. The 2011 government evaluation of the Health Extension Program found that only 29% of health posts had functional refrigeration, confirming the extent of the problem [5]. The fact that less than half of HEWs learned about these tasks during pre-service education likely contributes to infrequent practice. Because HEWs are the only ones available to undertake these essential tasks at health posts, both pre-service education and onsite mentorship should be redesigned to address them.

Other tasks performed infrequently, such as participating in research activities focusing on data management and reporting, may reflect HEWs lack of appreciation of the task’s importance; almost one-fifth of providers did not rate this task as highly critical for health outcomes. This suggests a failure of pre-service education to explain the importance of the task. It is crucial for HEWs to learn how to generate community based information and record data in order to make informed decisions about community health issues and concerns.

Maternal and child health morbidity and mortality are priority issues for Ethiopia [23] and improving these indicators were one of the reasons for developing the HEW workforce. However, the results of this task analysis suggest that weaknesses in training, including confusion over whether tasks are supposed to be taught during pre-service or in-service training, may be undermining HEWs’ ability to offer these services. Fully 22% of respondents did not feel competent to provide Integrated Management of Newborn and Child Illness (IMNCI) and the same percentage had never received any training on the task. This finding is consistent with the government evaluation of the Health Extension Program [5], which reported poor competency on IMNCI. Together these findings warrant the revision of the pre-service education curriculum to enhance HEWs’ knowledge and skills in this area. Moreover, offering in-service and on-the-job training and mentorship for HEWs may be crucial because this task targets common causes of child morbidity and mortality and it is one of the important child survival strategies for low-income countries, including Ethiopia [24].

There were similar findings related to the performance of childbirth services: 22% of HEWs rated themselves as not competent and 16% were never trained to perform the task even though it is part of their scope of practice. Both the national program evaluation [5] and a regional performance evaluation [9] noted that most HEWs assisted births but their knowledge and performance was poor. According to a systematic review of ANC and postnatal care services in low-income countries, there has been a substantial increase in the percentage of pregnant women attending ANC since the HEW program began in Ethiopia, but HEWs have had little impact on the use of skilled birth attendants [18]. It may be that neither pre-service education nor in-service/on-the-job-training were adequately preparing most HEWs to perform this task. This task should be a training priority, and it may be important to emphasize strengthening the capacity of HEWs with regular mentorship and in-service training.

Lastly, this study raises questions about the breadth of HEWs’ scope of practice [6]. Multiple competing demands on HEWs’ time and skill set may help explain the deficits identified by the task analysis, including the number of tasks performed infrequently (especially health education and communication tasks), the number of tasks that pre-service education programs do not address, and HEWs’ inability to perform certain tasks. The 2011 evaluation of the Health Extension Program concluded that engaging HEWs in such a wide range of tasks negatively affected their performance [5]. This problem is not limited to Ethiopia. For example, Health Surveillance Assistants in Malawi, who fulfill a similar role to HEWs, complained about being overloaded and unable to perform their jobs properly after they were assigned additional tasks [25]. However, the problem may be more acute in Ethiopia, because HEWs have a more extensive job description than any other full-time, salaried multipurpose health workers operating at the larger community level [22]. The findings suggest that HEWs’ scope of practice should be reviewed and possibly narrowed.

Based on the data collected by this study, it is not possible to determine why so many tasks in HEWs’ scope of practice were not covered in pre-service education. Possibilities include that: the tasks were not part of the curriculum, there was insufficient time during training to cover all tasks in the curriculum, or skills labs and clinical practice opportunities were lacking for certain tasks. It was also beyond the scope of this study to explore why HEWs performed certain tasks infrequently, which could be related to other issues such as lack of infrastructure. Additional research is needed to explore these issues.

### Strengths and limitations

This study is the first of its kind to look at the practice of HEWs in Ethiopia using task analysis research methodology. Strengths of this study are the inclusion of HEWs from rural health posts across the five most populous regions of the country, the generation of data that reflect the practice frequency of selected tasks using a proven research method, as well as information about education and performance. Though using small sample size, study findings offer valuable insights into priorities for strengthening the education and practice of this critical cadre. There could be sample selection bias, but we believe that it was minimal because the study was supported by the FMOH, provided written sample selection procedures to regional health bureaus and was not an evaluation of health extension program performance in the sample health posts. Self-reported competence instead of objective assessment could also be a limitation. Social desirability and recall bias may also affect the responses. Attempts to reduce social desirability bias included careful explanation of the purpose of the study and the anonymity of the responses and the de-identifications of study participants.

## Conclusions

Pre-service education did not prepare HEWs for all the tasks that comprise their scope of practice. Over half of HEWs learned these tasks after graduation, through in-service or on-the-job training. HEWs perceived themselves as sufficiently competent to perform most tasks, with the notable exception of IMNCI, institutional deliveries, and management of supplies, stocks and maintenance at facilities, all of which are key parts of national strategic health plan. These deficits may limit the effectiveness of the Health Extension Program to provide preventive and basic curative health services in communities. While few similar studies are available from other settings, the limited information available suggests that community based health worker programs in other low-income countries may face similar problems and could benefit from this kind of task analysis for improving universal access to primary health care services.

It is important for the entire scope of HEW practice to be included within the pre-service curriculum in Ethiopia in order to avoid the expense of repeating this education and training at different sites following graduation. The Ministry of Health has already taken steps to extend HEW training by launching a continuing professional development scheme that provides an additional year of education for HEWs who upgrade their credentials. A review of HEW curricula that focuses on expected practice informed by results from this study would identify gaps in the curricula as well as duplications and outdated content. In-service training and professional development can then be targeted more precisely to the identified needs of HEWs already in practice. Further, a review of the HEW scope of practice may highlight areas that should be prioritized or deemphasized to ensure that the breadth of required practices is manageable. Continuing mentorship and supervision are also important for HEWs to ensure the quality of health services provided to communities.

## Abbreviations

ANC: Antenatal Care
FMoH: Federal Ministry of Health
HEWs: Health Extension Workers
ICCM: Integrated Community Case Management
IMNCI: Integrated Management of Newborn and Child Illness
PMTCT: Prevention of Mother-to-Child Transmission
PSE: Pre-Service Education
SNNP: Southern Nations, Nationality and People
WHO: World Health Organization
